# Fractal analysis of peripheral blood neutrophil chromatin in patients infected with *Trypanosoma cruzi* and/or HIV correlates with left ventricle ejection fraction, T CD4+, and T CD8+ lymphocytes levels

**DOI:** 10.1590/1414-431X2025e14940

**Published:** 2025-10-17

**Authors:** N.M. Silva, G.S.M. Porto, A.E. Carvalho, N.P.M. Leite, C.M. Andrade, T.A.A.M. Fernandes, V.D. Almeida, J.S.O. Filho, I.M.S.S. Lopes, T.S. Fernandes, M.F. Andrade

**Affiliations:** 1 Programa de Pós-graduação Stricto Sensu em Saúde e Sociedade, Universidade do Estado do Rio Grande do Norte, Mossoró, RN, Brasil; 2 Programa Multicêntrico de Pós-Graduação Stricto Sensu em Bioquímica e Biologia Molecular, Universidade do Estado do Rio Grande do Norte, Mossoró, RN, Brasil; 3 Departamento de Ciências Biomédicas, Universidade do Estado do Rio Grande do Norte, Mossoró, RN, Brasil; 4 Programa de Pós-Graduação Multicêntrico Stricto Sensu em Ciências Fisiológicas, Universidade do Estado do Rio Grande do Norte, Mossoró, RN, Brasil; 5 Departamento de Biofísica e Radiologia, Universidade Federal de Pernambuco, Recife, PE, Brasil; 6 Departamento de Ciências da Saúde, Universidade Federal Rural do Semiárido, Mossoró, RN, Brasil

**Keywords:** Chagas disease, HIV, Fractal analysis, Neutrophil chromatin

## Abstract

Fractal analysis (FA) of neutrophils has demonstrated potential in identifying changes in chromatin associated with clinical parameters in individuals with chronic diseases. Therefore, this study aimed to investigate FA of neutrophils' nuclei from patients with *Trypanosoma cruzi* and/or HIV. Fifty-three individuals were recruited and divided into four groups: *T. cruzi*-infected patients with chronic chagasic cardiomyopathy (CCC) (n=18), seropositive HIV individuals (SPHIV) (n=14), *T. cruzi*-HIV coinfected patients (n=9), and healthy individuals (n=12). Micrographs of neutrophils underwent FA using a box-counting method in the ImageJ software. Clinical parameters obtained from patients' medical records, such as left ventricle mass index (LVMI), left ventricle ejection fraction (LVEF), risk of ischemic stroke (IS), and sudden death were analyzed. FA was lower in patients compared to the control group (P<0.0001). Chagas disease (CD) patients showed higher FA when the LVEF was higher (r=0.53), which increased the risk of sudden death (r=-0.62). In SPHIV, when FA was higher, T CD4+ lymphocyte count was also higher (r=0.66) and the T CD8+ lymphocyte count was lower (r=-0.54). Coinfected individuals showed higher FA, when LVEF (r=0.60), neutrophil to lymphocyte ratio (r=0.80), total lymphocytes (r=0.70), and T CD4+ lymphocyte count (r=0.70) were increased, and T CD8+ lymphocyte count was decreased (r=-0.70). FA was an independent marker of changes in neutrophil chromatin and has proven to be a prognostic tool and a method for risk stratification for adverse events, survival, and mortality in individuals infected with *T. cruzi* and/or HIV.

## Introduction

The conventional Euclidean mathematical definition of dimension assumes that objects are homogeneous and uniform (1). According to this model, a line has dimension 1, a plane has dimension 2, and a solid has dimension 3. In nature, however, objects have complex and irregular shapes whose dimensions do not fit into the Euclidean model and have dimensions “between” the known values (1). Therefore, the term “Fractals” was introduced by Mandelbrot as a model to describe shapes, objects, and processes in different areas of science (2-4).

Fractals can be considered repetitions of the same shape that together compose a whole resembling its parts (4). In other words, they present a pattern of repeated structures observed at different levels of magnification. In nature, fractal patterns appear in different contexts, which, unlike computational models, are self-similar within a limited range (1).

Studies in the last 30 years have revealed fractal characteristics in cell biology and chromatin organization. The evaluation of these features can be easily measured in digitalized microscopic images and recent research has pointed out their potential value in diagnosis and prognosis of a number of clinical conditions (3). The fractal model can describe the complexity of macroscopic and microscopic anatomical structures such as the brain (5), lung (6), and gastrointestinal system (7). This pattern is also noticed in biochemical (8) and pharmacological (9) research.

Fractal analysis (FA) is the study of fractal patterns, with fractal dimension (FD) being a morphometric feature that quantifies the space occupied by a fractal set, taking into account the complexity of image contours, space availability, and irregularity of complex bodies (10). These analyses can be used to explore the behavior of complex systems and the adverse situations that can interfere and disrupt the order of not only physical but also biomedical systems, enabling researchers to infer the health status of individuals and their tissues (11). FD can be a strategy for measuring contours, surface areas, and other dimensional parameters of tissues and cells, including the assessment of cellular structures at specific functional or pathological stages (12). Since the first proposition to use FA to observe chromatin organization by Takahashi in 1989 (doi: 10.1016/s0022-5193(89)80012-8), many researchers have found associations of FA with different types of cancer, for example (13).

An important health issue that is occurring with the urbanization of Chagas disease (CD) is the coinfection with HIV in both endemic and non-endemic areas. The state of this coinfection is underreported (14) and co-infected patients have a high mortality rate, mainly due to the potential risk of reactivation because of immunosuppression (15). There is a high risk of the central nervous system being affected by *T. cruzi* (74.2%), followed by myocardial involvement (16.7%) (16).

The state of immunosuppression also modifies neutrophils, which are crucial in combating opportunistic infections. One of their most effective mechanisms is the release of neutrophil extracellular traps (NETs). The release mechanisms of NETs are distinct contributing factors to the emergence of nuclear alterations in neutrophils (17).

FD has been employed in the investigation of cellular modifications in various cell profiles and pathological contexts, including cancer (1,3). This has paved the way for research into neutrophil chromatin alterations in other clinical settings, including infectious states (18).

Although some emerging studies have investigated alterations in FA of peripheral blood cells (19,20), FA of neutrophils has only recently been used for the first time, uncovering distinct fractal patterns in the assessment of chromatin from metamyelocytes, rod-shaped cells, and segmented neutrophils from the peripheral blood of healthy individuals. FA demonstrated its efficacy as an independent parameter in determining neutrophil types in healthy subjects (21).

Currently, the tools available to assess the clinical evolution of CD complications are limited to serology tests, x-rays, electrocardiograms, Holter, and echocardiograms, which can be invasive, difficult to access, inconclusive, and non-predictive (22). Nonetheless, parameters from those examinations, notably left ventricle ejection fraction (LVEF) and Rassi scores, are still considered to be the best way to evaluate the health of patients. LVEF is a particularly good parameter to evaluate CD patients, being crucial in their prognostic stratification and also a strong mortality predictor, indicating ventricular dysfunction (23,24). The Rassi score is a method specially developed to evaluate the risk of death in patients with CD (25).

Considering that CD is an important risk factor for HIV co-infected patients, who may have the virus reactivated due to immunosuppression (14,15), there is a need for tools and biomarkers capable of providing consistent clinical monitoring. It is of great importance that these tools can be used in an easy non-invasive way and have high prognostic value, helping to predict clinical complications, make therapeutic decisions, and evaluate the effectiveness of therapeutic treatments in a timely manner (26).

Therefore, the aim of this study was to investigate FD as a marker for changes in the chromatin of neutrophils in patients infected with *Trypanosoma cruzi* or HIV and in *T. cruzi*-HIV co-infected patients. These represent three infectious states that each carry a high risk of both morbidity and mortality (27).

## Material and Methods

### Study design, population, and ethical considerations

This cross-sectional study recruited 53 individuals divided into four groups: patients seropositive for CD and HIV-negative (n=18); patients seronegative for CD and HIV-positive (SPHIV) (n=14); patients seropositive for CD and HIV-positive (n=9); and control group, seronegative for CD and HIV-negative (n=12). The sample was non-probabilistic and intentional and all participants lived in municipalities in the mesoregion of Western Potiguar, in the State of Rio Grande do Norte (Brazil). The control group included 6 males and 6 females, the CD group, 9 males and 9 females, the co-infected CD-HIV, 7 males and 2 females, and the SPHIV, 11 males and 3 females. The mean age of participants in the control group was 45.33 (±9.56), that of the CD was 58.33 (±9.02), that of the co-infected CD-HIV was 50.78 (±9.82), and that of the SPHIV was 43.50 (±14.59).

CD patients were clinically monitored at the CD Outpatient Clinic of the State University of Rio Grande do Norte (UERN). SPHIV patients were monitored at the Rafael Fernandes Hospital (Mossoró, Brazil), which is a reference institution for treating patients with infectious diseases. Co-infected patients were followed up in both health service centers. All CD patients were symptomatic and diagnosed with CCC (chronic chagasic cardiomyopathy) while co-infected CD-HIV, presented with cardiac involvement as a consequence of Chagas disease, but were not diagnosed with CCC.

To confirm *T. cruzi* infection, at least two different serological tests were used (Chagatest recombinant ELISA v. 4.0 - Wiener; HAI - Wiener; or Indirect Immunofluorescence Assay - ImunoCON Chagas - WAMA), as recommended by the World Health Organization (28). HIV infection was confirmed by two rapid serological tests (Cepheid, USA). All HIV-positive patients underwent viral load assessments (Abbott Real Time, USA) and counts for T CD4+ and T CD8+ lymphocytes by BD TrucountTM kit (BD Biosciences, USA) (29). The control group was composed of individuals testing negative for both CD and HIV. Individuals from all groups with a current medical history of other comorbidities such as infectious diseases, cancer, systemic inflammatory diseases, bacterial infections, antibiotic usage for at least two months, or current treatment with glucocorticoids were excluded.

The Research Ethics Committee for Human Subjects at the State University of Rio Grande do Norte, Mossoró, Brazil, approved this research under protocol number 2.672.657.

### Neutrophil-to-lymphocyte ratio

Patients' whole blood samples were collected into tubes containing the anticoagulant EDTA. The neutrophil-to-lymphocyte ratio (NLR) was derived from the total cell counts undertaken by a partner institute, the Clinical Analysis Laboratory of Rafael Fernandes Hospital (Mossoro, RN), using a BC-3000 Plus automated blood cell counter (Mindray Corp., China).

### T CD4+ and T CD8+ lymphocyte counts, TCD4+/TCD8+ ratio, and viral load quantification in HIV-positive patients

An aliquot of 4 mL from each blood sample was transported to the Central Laboratory of the State of Rio Grande do Norte, Brazil. The T CD4+ and T CD8+ lymphocyte counts were carried out using flow cytometry, employing the BD Trucount Tubes kit (USA). The TCD4+/TCD8+ ratio was calculated by dividing the T CD4+ lymphocyte count by the T CD8+ lymphocyte count. Viral load quantification was determined using a real-time PCR technique.

### Cardiological assessment and risk score stratification for stroke and death in patients with CD

The risk stratification for ischemic stroke due to cardioembolic causes (30) and the risk of sudden cardiovascular death in patients with Chagas cardiomyopathy (25) were undertaken using supplementary tests and data retrieved retrospectively from medical records, with prior authorization from the patients. These tests included an electrocardiogram, 24-h Holter monitoring, transthoracic echocardiogram (ECOTT), and chest X-rays (postero-anterior and lateral views). Given the operator-dependent nature of the ECOTT imaging test, the same cardiologist specializing in echocardiography examined all patients. LVEF was obtained from echocardiography data recorded in the medical records of all patients.

### Chromatin imaging from peripheral blood neutrophils

Routine venipuncture blood smears were prepared, stained with Leishman dye, and examined at ×1000 magnification with a 16-megapixel camera attached to an Olympus BX41 microscope (Japan) with 75% luminosity (21). One hundred neutrophils per individual (5,300 in total from all subjects) were documented through micrographs using a validated computational routine specific for neutrophil processing (30).

The images were acquired in JPEG format due to its compression efficiency. Although this format uses lossy compression, it does not compromise the analysis, as both control and test images were acquired under the same conditions. Sharpness and contrast enhancement were standardized according to the guidelines established by the responsible team, following visual standardization principles to ensure a consistent appearance across all analyzed images. Additionally, adjustments were made to improve the readability of key elements and correct potential technical defects introduced during image acquisition or processing. The main objective was to minimize the introduction of artifacts that could compromise the analysis or interpretation of the images. After acquisition and processing, the final result focused exclusively on the object of interest i.e., nucleus structure.

### Image processing

The acquired images underwent a nucleus isolation process, enabling the separation of the object of interest (nuclear region occupied by chromatin) from other cellular components. For this purpose, the Paint software (Microsoft Windows, version 6.1, USA) was used. Subsequently, in the ImageJ software (NIH, USA), images were converted into grayscale (8 bits). The image calculator was applied to sharpen the images, accentuating the chromatin texture by increasing brightness and contrast, followed by a standardized conversion to grayscale tones for all micrographs. After this processing, FA analysis was performed (18,31).

### Fractal analysis

The value of FD was obtained using the Plugin “FracLac” incorporated in the ImageJ software. The pre-set was adjusted to evaluate the image in grayscale mode. The software automatically calculates the overlay of boxes in the image. The side of these boxes continually decreases in size, producing a reduction number (r). With each of these reductions, the number of boxes needed to cover the image pixels (Nr) is tallied (31). Consequently, the FD of the chromatin is the slope of the regression line generated by the logarithm of the number of boxes as a function of the logarithm of the box sizes, according to the following equation:

$$FD=-\underset{r\to 0}{\lim}\left(\frac{\log Nr}{\log\left(\frac{1}{r}\right)}\right)$$ (Eq. 1)

Based on the intensity range of the pixels, FracLac determines a threshold value to distinguish the object of interest (chromatin) from the background (nuclear regions without chromatin) (20,32) and thereby generating a FD value for each cell nucleus.

### Statistical analysis

GraphPad Prism software (version 8.0.2, USA) facilitated the data processing, and a 95% confidence interval was used. The Shapiro-Wilk test was used to assess data normality. For variables with a normal distribution, the Student's *t*-test was used (for comparing two variables), and ANOVA was followed by the Tukey test (for multiple comparisons). For variables with a non-normal distribution, the Mann-Whitney test was applied (for comparing two variables) and the Kruskal-Wallis test followed by Dunn's test (for multiple comparisons). The Levene test was used to verify the variance of the FA among study groups. The correlational analysis between the FA and clinical parameters was made with the Pearson test (for parametrically distributed variables) and the Spearman test (for non-parametrically distributed variables).

## Results

### FD of neutrophil chromatin from patients infected with *T. cruzi* and/or HIV

**Figure 1:**
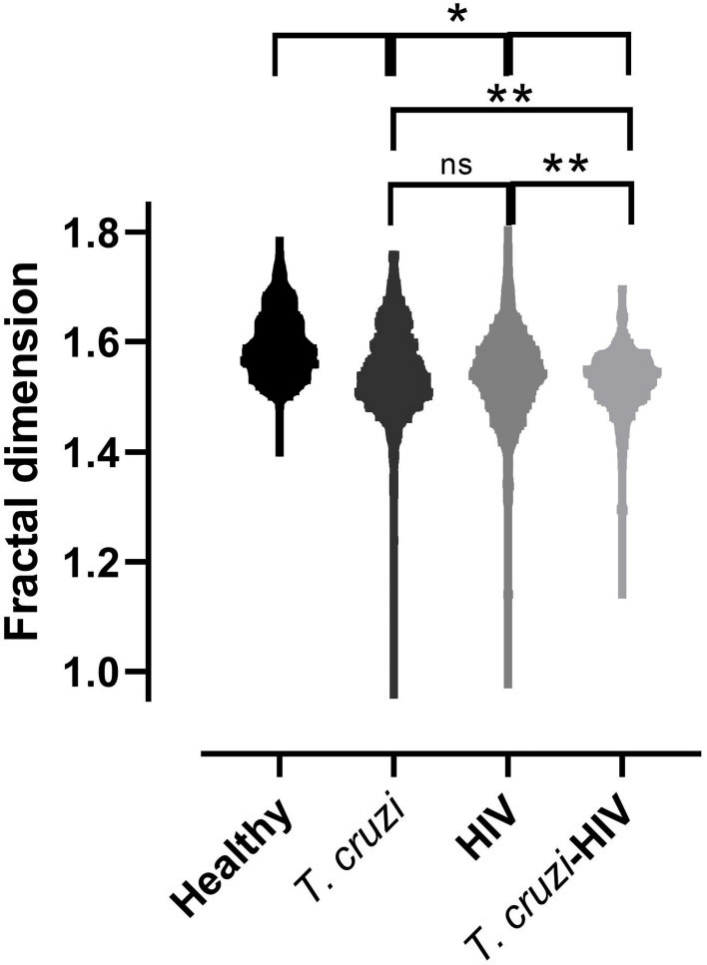
Analysis of fractal dimension of chromatin in neutrophils from healthy individuals and those infected with *T. cruzi* and/or HIV. *P<0.001, **P<0.0001. Kruskal-Wallis followed by Dunn's multiple comparisons test. ns: not significant.

Data in Figure 1 indicate that individuals infected with *T. cruzi* and/or HIV exhibited a FA of neutrophil chromatin distinct from that found in healthy individuals (P<0.0001). A difference was also found between the co-infected *T. cruzi*-HIV patient group and both the patients with CD (P<0.0001) and those only HIV-positive (P<0.0001). However, no difference was found between the *T. cruzi* group and the HIV group (P>0.9999).

In the multiple-group comparison, the Levene test indicated that the variances were not homogeneous (P<0.0001). Thus, there was a high heterogeneity in the FA among the infected groups compared to the healthy group.

### FA and clinical characteristics of *T. cruzi*-infected patient

**Figure 2:**
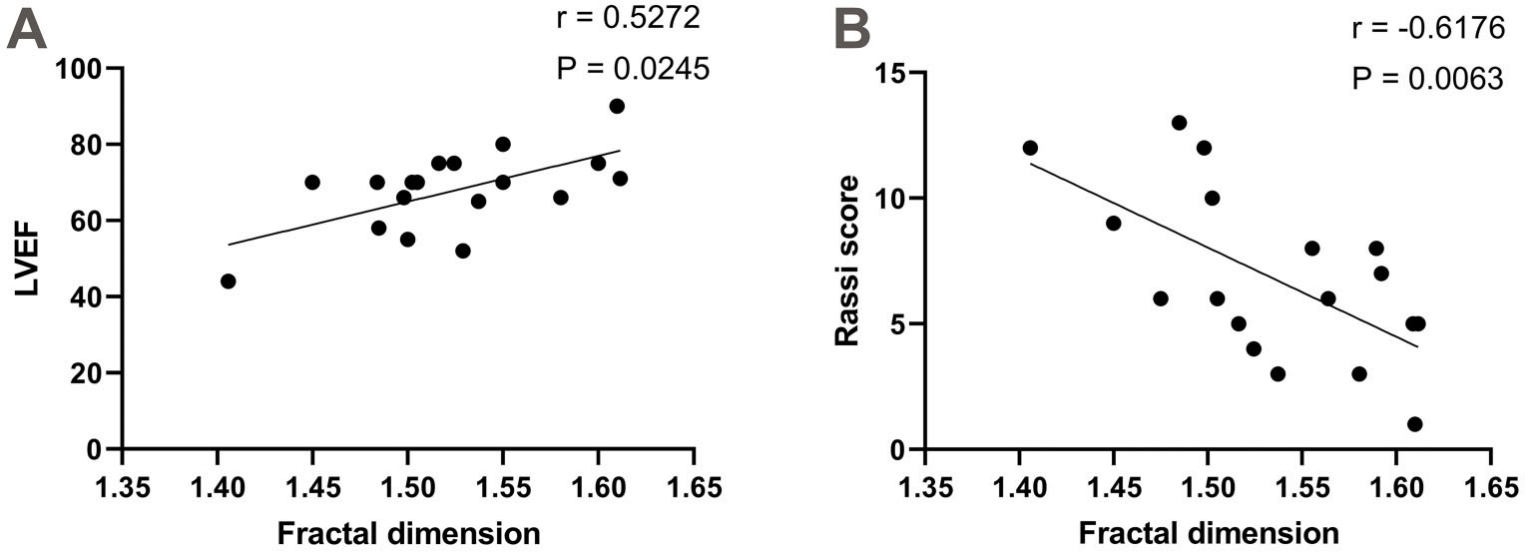
Spearman correlation between left ventricular ejection fraction (LVEF) and Rassi score and chromatin fractal dimension in neutrophils from peripheral blood of *T. cruzi*-infected patients. Patients were grouped by the Rassi score into low (n=10), intermediate (n=4), and high (n=4) risk of death from cardioembolic causes.

Significant correlations were identified between FA, LVEF (r=0.5272; P=0.0254, 95%CI: 0.06520 to 0.8031), and the Rassi score in patients with CCC (r=-0.6176; P=0.0063, 95%CI: -0.8460 to -0.1974) (Figure 2). When the FA of neutrophil chromatin was higher, LVEF also increased. Conversely, assessed by the Rassi score, the risk of sudden death in patients with CD increased as the FA decreased. The other variables did not show a significant correlation with FA: total neutrophils (P=0.0810), total lymphocytes (P=0.4884), NLR (P=0.9708), LVMI (P=0.2466), and risk of ischemic stroke (P=0.9628).

**Figure 3:**
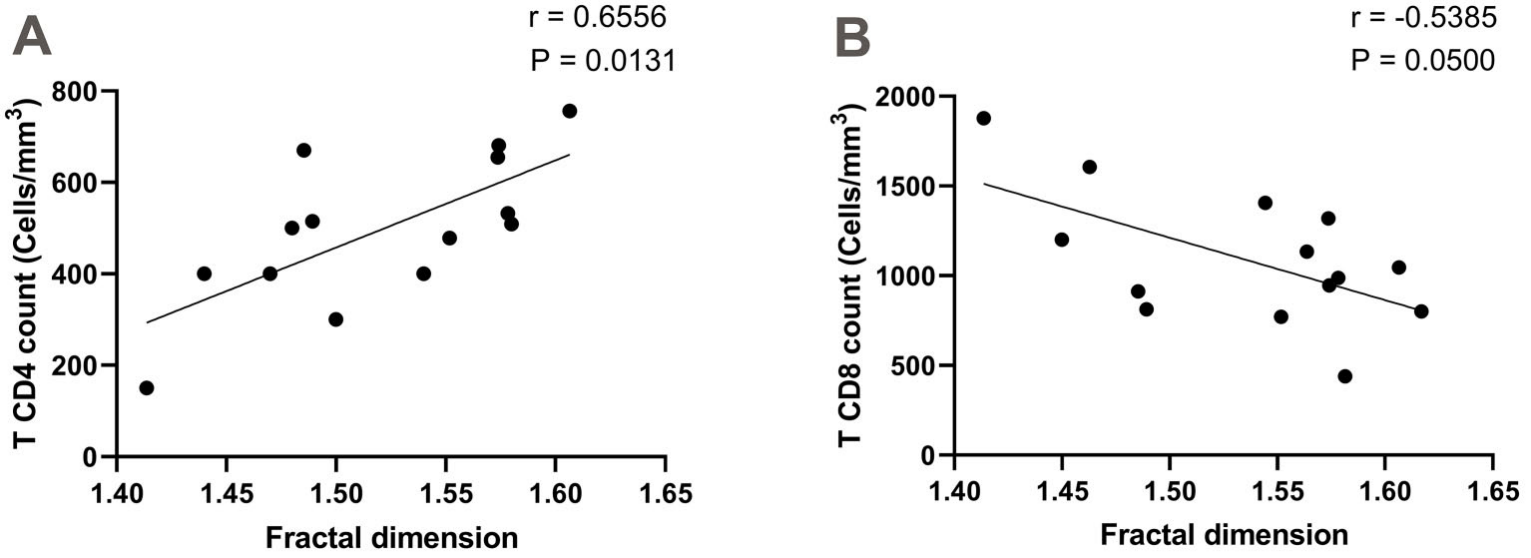
Spearmen correlation between CD4+ and CD8+ T lymphocyte counts and chromatin fractal dimension of neutrophils from peripheral blood of individuals with HIV infection only (HIV-positive).

In the SPHIV group, a positive correlation was found between FA and T CD4+ lymphocyte counts (r=0.6556; P=0.0131, 95%CI: 0.1749 to 0.8839) and a negative correlation between FA and T CD8+ lymphocyte counts (r=-0.5385; P=0.0500, 95%CI: -0.8368 to 0.006435) as shown in Figure 3. No significant correlation was observed between FA and the following variables: total peripheral blood neutrophils (P=0.7500), total peripheral blood lymphocytes (P=0.9758), NLR (P=0.6051), and T CD4+/T CD8+ ratio (P=0.9758).

**Figure 4:**
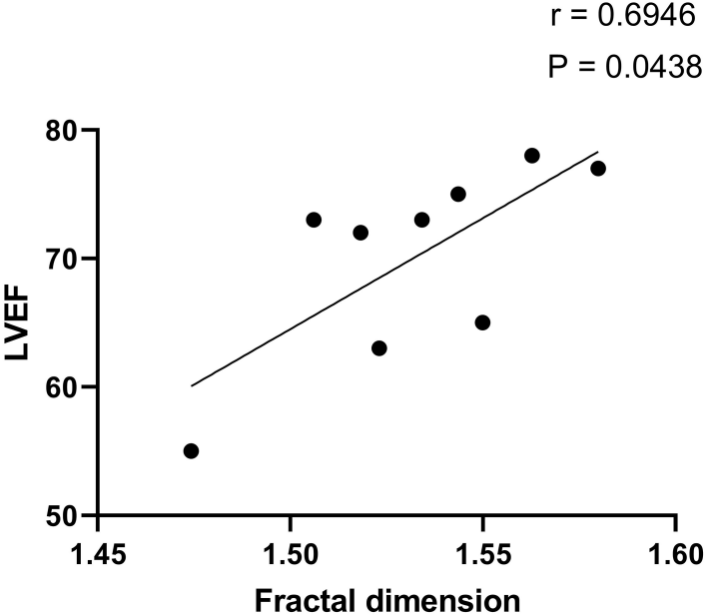
Correlation between the fractal dimension of peripheral blood neutrophil chromatin and left ventricle ejection fraction in individuals with *T. cruzi*-HIV co-infection.

**Figure 5:**
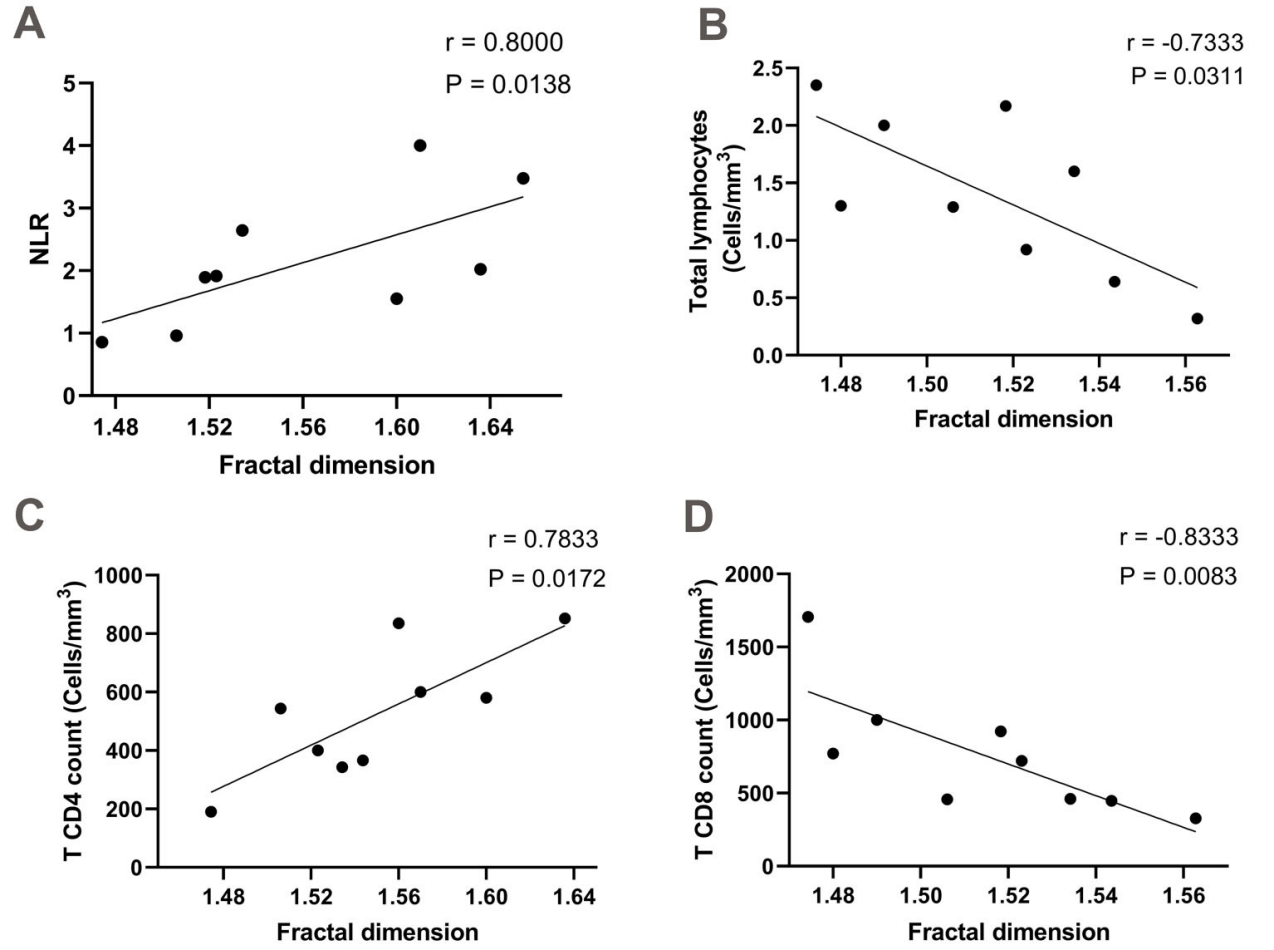
Spearman correlation between the fractal dimension of peripheral blood neutrophil chromatin and laboratory parameters in individuals with *T. cruzi*-HIV co-infection. Neutrophil/lymphocyte ratio (NLR) and total lymphocytes were measured using the BC-3000 Plus automatic counter. The CD4+ and CD8+ T lymphocyte count was performed by flow cytometry.

For the co-infected group (*T. cruzi*-HIV), a correlation was found between FA and LVEF (r=0.6946; P=0.0438) as presented in Figure 4. Figure 5 shows other significant correlations with NLR (r=0.8000; P=0.0138), total lymphocytes (r=-0.7333; P=0.0311), T CD4+ counts (r=0.7833; P=0.0172), and T CD8+ counts (r=-0.8333; P value: 0.0083). No correlation emerged between FA and total neutrophil count (P=0.1080), T CD4+/TCD8+ ratio (P=0.1475), and LVMI (P=0.3388).

## Discussion

The results of the FA showed alterations in neutrophils' chromatin from individuals with CD, seropositivity for HIV, and *T. cruzi*-HIV coinfection compared to the healthy control group. When comparing the FA among infected groups, there was no difference between the *T. cruzi*-infected group and the SPHIV group. However, there was a difference between these two groups and the *T. cruzi*-HIV co-infected group.

Furthermore, significant variability was found in nuclear FDs across all infected groups. These findings may suggest the presence of neutrophil subtypes, which could play pivotal roles in the pathophysiology of the cardiac form of CD (16), as well as in HIV infection and in the *T. cruzi*-HIV coinfection. To validate this hypothesis, it is essential to investigate these potential subpopulations through genetic and immunophenotyping tests and develop experimental assays capable of culturing neutrophil subtypes and evaluating their effector responses.

A reduction in FA in patients infected solely by *T. cruzi* correlated significantly with decreased LVEF and increased risk of death. Reduced LVEF is known to indicate the onset of heart failure, becoming one of the most crucial predictors of death in patients with CCC (25,31,33,34). Conversely, the risk of death showed a negative correlation with FA. The risk of sudden death from cardioembolic causes, as proposed previously (35), provides the highest predictive value in risk of death stratification of patients with CD presenting the cardiac clinical form (36). These data suggest that the FA of chromatin in peripheral blood neutrophils from patients with CD might serve as a potential biomarker for disease prognosis.

In patients infected solely by HIV, FA positively correlated with T CD4+ lymphocyte count and negatively with T CD8+ lymphocyte count. This correlation was observed in *T. cruzi*-HIV co-infected patients as well. Regarding this uncommon coinfection, this study has demonstrated FA's potential to correlate with a crucial clinical parameter of patient monitoring and prognosis: the reduction in LVEF, even in patients without CCC, which applies to the co-infected patients. These findings suggest that a reduced nuclear FA indicates a poorer prognosis and higher mortality in co-infected patients because the lower the FA of chromatin in peripheral blood neutrophils, the lower the LVEF and T CD4+ lymphocyte count. Although these patients did not have a defined clinical form due to the absence of complementary clinical examinations, they had some type of cardiac involvement. This enabled their results to be compared with patients having the cardiac clinical form of CD.

A positive correlation was found between FA and the NLR and a negative correlation was found between FA and the total lymphocyte count. In this patient group, a reduced FA and, consequently, reduced cellular maturity (18) might be among the events involved in the pathophysiology of these complications. Conversely, increased cellular maturity (higher FA) influences cardiovascular performance (higher LVEF) and is associated with a greater likelihood of immunological recovery, with rising T CD4+ lymphocyte counts. Such evidence points to FA's potential as a valuable prognostic tool in managing patients infected by *T. cruzi* and/or HIV.

For the first time, the results presented in this study indicated the potential of employing FA as a marker for nuclear changes in peripheral blood neutrophils, suggesting FA's possible use in the clinical monitoring of patients with CD and/or HIV. This could assist in patient prognosis by identifying risks for adverse clinical events and stratifying individuals by survival and mortality.

The findings of this study support the feasibility of utilizing FA in a medical care context and align with similar perspectives found in other studies that employed fractal geometry for recognizing changes in medical images, diagnosing pathologies such as prostate, breast, and lung cancer and hematological diseases, and determining their prognosis (3,19,31,32,35,37-40).

This is the first study to reveal that the FA of chromatin from neutrophils in the peripheral blood of patients infected with *T. cruzi* and/or HIV is lower than that observed in healthy individuals. Furthermore, correlations between FA and clinical parameters suggest a potential association between a reduced FA and unfavorable clinical outcomes in patients with CCC, in those infected with HIV, and in carriers of the *T. cruzi*-HIV coinfection. This offers promising avenues for employing the FA of neutrophil nuclei as a biomarker for the prognosis of these chronic infectious conditions.

Greater technical knowledge concerning FA and subsequent cohort studies are necessary for elucidating the mechanisms implicated in the associations of neutrophils and parameters of CD, HIV infection, and *T. cruzi*/HIV co-infection.
